# Effect of continuity of care on health-related quality of life in adult patients with hypertension: a cohort study in China

**DOI:** 10.1186/s12913-016-1673-2

**Published:** 2016-11-28

**Authors:** Ting Ye, Xiaowei Sun, Wenxi Tang, Yudong Miao, Yan Zhang, Liang Zhang

**Affiliations:** 1 School of Medicine and Health Management, Tongji Medical College, Huazhong University of Science and Technology, 13 Hangkong Road, Wuhan, China; 2 School of International Pharmaceutical Business, China Pharmaceutical University, Nanjing, China

**Keywords:** China, Continuity of care, Hypertension, Health-related quality of life, Cohort study

## Abstract

**Background:**

Continuity of care is widely considered a principle of primary care that decreases healthcare utilization and mortality. However, the effect of continuity of care on health-related quality of life (HRQoL) for adult patients with hypertension remains unclear.

**Methods:**

To further evaluate the effect of continuity of care, we implemented a cohort study among hypertensive patients aged over 35 years (*n* = 1200) in six townships in Qianjiang District, Chongqing, China, between 2012 and 2014. The study ultimately included 1079 participants. The continuity of care index was calculated using claim-based longitudinal data obtained from hypertension follow-up service records. The baseline and endline survey-based data, tested by the SF-36 scale, were used to assess HRQoL. To control selection bias and examine the effect of continuity of care, a kernel-based propensity score matching difference-in-differences (DID) method was used. Additionally, descriptive statistics, chi-squared test, and Mann–Whitney nonparametric test were used to summarize characteristics, evaluate proportional differences, and analyze statistical differences, respectively.

**Results:**

Our results showed that patients in the high continuity of care group presented greater improvement in both Physical Component Summary (PCS, DID = 5.192 ± 1.970, *p* < 0.001) and Mental Component Summary (MCS, DID = 7.900 ± 1.815, *p* = 0.008) than those in the low continuity of care group. Moreover, patients in the high continuity of care group showed significant improvement in physical functioning, role-physical, general health, role-emotional, and mental health.

**Conclusions:**

Our findings indicate that a long-term physician-patient relationship may improve HRQoL in patients with hypertension. However, more unified measurement tools are needed to evaluate continuity of care. Further studies should include more study settings.

## Background

Continuity of care is defined as a core attribute of primary care. However, considerable diversity exists in previous definitions. For instance, Saultz [1] defined continuity of care as a hierarchical concept that ranges from informational continuity and longitudinal continuity to interpersonal continuity. In his study, longitudinal continuity emphasized a familiar setting for patients to receive care that is easier for patients to access; interpersonal continuity was characterized by trust and a sense of responsibility. Nevertheless, Haggerty [2] combined longitudinal continuity and interpersonal continuity into one concept, called relational continuity. This study focuses on a long-term relationship for patients and physicians, without involving the sense of trust and responsibility. Additionally, this type of continuity of care is the same as the longitudinal continuity suggested by Saultz [1].

The effects of continuity of care have been debated in previous studies because some have concluded that continuity of care is associated with fewer hospitalizations, emergency department visits, and hospital admissions [3–7]; low pharmaceutical expenditures and healthcare expenses [5, 8–10]; decreased mortality rates [11, 12]; fewer duplicated medications [13]; improved medication adherence [14]; and patient satisfaction [15–18]. However, others insist that high continuity of care may lead to the purchase of more drugs overall and that the effects on HRQoL are unclear; thus, these should be further examined [19, 20].

Regarding the association between continuity of care and HRQoL, Hanninen [21] reported that diabetic patients who had been treated by the same general practitioner for at least 2 years seemed to have better mental and physical health, as well as less pain, than those who did not have a long-term physician-patient relationship. However, as pointed out by Hanninen [21], a causal relationship could not be established because many confounding factors were not controlled by the cross-sectional investigation. Meanwhile, Gulliford [22] implemented a cohort study for diabetic patients using random effect models adjusted for baseline value of outcome, age, sex, ethnicity, duration of diabetes, type of treatment, qualifications, housing tenure, and living alone. Similar to Gulliford’s study, we also attempted to reduce the confounding bias and find a causal relationship between continuity of care and HRQoL among patients with hypertension. In contrast to the study by Gulliford, a kernel-based propensity score matching DID analysis method was presently used to match the high continuity of care group and the low continuity of care group, yielding comparable treated and control groups of patients with hypertension.

Hypertension is one of the main chronic diseases in China [23]. A 2014 report notes that the prevalence rate of hypertension is 29.6 % for persons aged 18 years or older [24]. In 2009, free health services for hypertension patients aged over 35 years were included in the national public health program, named the National Essential Public Health Services Package (NEPHSP). This program establishes health records, screening, following-up, and systematic physical examinations for hypertensive patients in urban community health centers and rural township health centers [25]. Moreover, the essence of having a family doctor lies on the principle of establishing a fixed relationship and continuity of care. The question that we aimed to answer is “Can a family doctor (representing continuity of care) improve HRQoL for patients with hypertension?” This question is important because the answer may influence the priority of basic public health reform that aims to sustain continuity of care and implement family doctor policies as compared to ensuring health service accessibility.

Therefore, to provide more empirical evidence of the effects of continuity of care and investigate its specific effects on HRQoL in China, we conducted this study to examine whether better continuity of care could improve HRQoL of hypertensive patients.

## Methods

### Setting and study sample

We selected Qianjiang District in Chongqing, China, as the study setting. The data analyzed herein were from the “Study on the Efficiency and Effectiveness of the Integrated Health Care Services in Rural China” funded by the China Medical Board, which was designed as a clustered randomized controlled trial in nearly 60 villages of six towns, with around 6000 chronic patients. Further details may be found in Tang’s paper [26].

To include patients with essential hypertension, the exclusion criteria for this study were as follows: (i) patients aged less than 35 years by December 2012, (ii) patients that were not registered as members of the NEPHSP program until January 2012. In the baseline investigation, conducted from July to August 2012, 1200 hypertensive patients were enrolled. The following patients were also excluded: (i) patients who did not use the stated address as their primary residence, (ii) those who received hypertension follow-up services less than three times a year, and (iii) those no longer requiring follow-up because of death. According to the above exclusion criteria, 121 patients were excluded, and 1079 patients were included in the study sample.

Given that hypertension patients were members of the NEPHSP program, these patients were followed up by physicians in the township health centers between January 2013 and December 2014. Ethical approval for this study was granted by the Ethics Committee of Tongji Medical College and Huazhong University of Science and Technology. All of the participants gave a written informed consent for participation in this study, provided consent before filling out the questionnaire, and consented to the publication of the data.

### Measures

#### Measure of continuity of care

In order to depict continuity of care patterns, patients were asked regarding their experience while attending physician consultations and their responses were recorded by physicians in the township health centers. These accounts were kept in each patient’s individual health record. When they visited or were called by their physicians in the township health centers, the trained physicians asked them whether they consulted other doctors for treatment of hypertension during two follow-up periods. If so, the information about those experiences was also recorded in each patient’s individual health record, including the name of the medical institution, the date of the visit, and the doctor’s name (if possible). All of the 1200 patients’ individual health records were collected between January and February 2015.

Various methods are used to measure continuity of care [27–31]. Additionally, most of the indices were classified into three types by Saultz [1]. These measures include those that do not require an assigned provider, measures that require an assigned provider, and measures of family continuity. Considering that assigned doctors or general practitioners are nonexistent in the Chinese health care system, designating a primary care physician for hypertensive patients is difficult. Therefore, to measure the concentration of visits with various providers we selected the continuity of care index [32, 33], which did not require a registry that assigned a physician for each patient and was the most used measure in prior research. This index is a dispersion measure that ranges from 0 (poorest continuity) to 1 (highest continuity), and can be computed as follows:

$$ \mathrm{Continuity}\ \mathrm{of}\ \mathrm{care}\ \mathrm{index} = \frac{{\displaystyle {\sum}_{i=1}^M}\kern0.5em {n_i}^2-N}{N\left(N-1\right)}, $$

where *N* represents the total number of visits, *n*_*i*_ is the number of visits to the same physician *i*, *i* is a given physician, and *M* is the number of physicians.

#### Measure of HRQoL

Patient HRQoL was assessed using the Medical Outcome Study Short-Form 36-Item Health Survey (SF-36 Scale) developed by the RAND Corporation’s Health Insurance Experiment [34]. The eight dimensions of HRQoL, the summary of physical quality of life (Physical Component Summary; PCS), and emotional quality of life (Mental Component Summary; MCS) were measured, as well as the reported health transition. The PCS and MCS were calculated by determining the mean average of all of the physically relevant questions (physical functioning, role-physical, body pain, and general health) and of all of the emotionally relevant items (vitality, social functioning, role-emotional and mental health), respectively [35, 36]. In the baseline and endline surveys, the data on HRQoL were collected through the SF-36 Scale.

### Other covariates

In the baseline investigation, we used a self-designed questionnaire to collect patients’ socio-demographic characteristics (age, sex, education level, and marital outcome), as well as information on the duration (years) of hypertension.

Considering that continuity of care may have some association with health care provision capacity, the six towns were divided into two groups, a high capacity group and a low capacity group. This grouping was based on multiple considerations on their performance in the most recent 5 years and consulting from the leaders of the Health Bureau in Qianjiang District. Additionally, because of the project “Study on the Efficiency and Effectiveness of the Integrated Health Care Services in Rural China”, two of the six towns implemented both care integration and payment integration interventions, two of them implemented care integration only, and the final two did not apply any intervention. The latter served as the control groups. In this study, the intervention types were also considered. In the final model, the interaction of health care provision capacity and hypertension intervention type were included.

### Statistical analysis

A propensity score matching DID approach was used. This approach isolates the improvement in outcomes related to the high continuity of care group that exceeds changes over the same period in the low continuity of care group. In this study, patients with continuity of care index = 1 were classified into the high continuity of care group. This group represented the treated group. Other patients were classified into the low continuity of care group, that is, the control group.

Propensity score matching was used to match the high continuity of care group and the low continuity of care group, so that the treated and control groups were comparable to avoid selection bias related to several key factors. To create the propensity score, a logistic regression model was created with the high continuity of care patients (vs. the low continuity of care patients) as the dependent variable. The sociodemographic variables (age, sex, education level, and marital outcome) and duration of hypertension were included as independent variables, as well as the interaction of health care provision capacity and type of hypertension intervention collected from baseline investigations. This matching ensured that the patients in the high continuity of care group and in the low continuity of care group were comparable.

Kernel-based propensity score matching was used. Covariate imbalance before and after matching was checked with the absolute standardized difference of the means of the linear index of the propensity score in the treated and (matched) non-treated groups (Rubins’ B) and the ratio of treated to (matched) non-treated variances of the propensity score index (Rubin’s R).

The DID analysis includes the weights derived from the kernel-based propensity score matching [37–40]. We performed a balancing test of the difference in the means of the covariates between the control and treated groups in the baseline period to test whether the parallel trends in the baseline period was satisfied, as this is one of the key assumptions of a DID methodology.

Descriptive statistics were used to summarize the patients’ baseline characteristics. The chi-squared test was used to evaluate proportional differences in categorical variables. The Mann–Whitney nonparametric test was used for between-group differences.

All analyses were conducted using Stata 13.0. We set statistical significance at a two-tailed *p* < 0.05.

## Results

### Baseline demographics

Of the initial 1079 hypertensive patients, 123 were in the high continuity of care group and the remaining 956 were in the low continuity of care group (Table 1). No significant differences were observed in the two groups regarding the distribution of age, sex, education level, marital outcome, duration of hypertension, and health care provision capacity of the township health centers. However, the distribution of types of hypertension intervention was statistically different. When comparing the dimensions of HRQoL, the patients in the high continuity of care group indicated worse scorings when compared to the ones in the low continuity of care group. Furthermore, statistically significant differences were found between patients in the scores of the following domains: physical functioning, role-physical, social functioning, role-emotional, mental health, and MCS.

**Table 1:** Patients’ characteristics in the high continuity of care group and low continuity of care group in the baseline period before matching (July–August 2012)

Variables	High continuity of care group	Low continuity of care group	*p*
Number of observations	123	956	-
Age (years), median (range)	70 (43–94)	69 (36–108)	0.659
Female, n(%)	64 (52.03)	530 (55.44)	0.475
Higher than primary educational level, n(%)	25 (20.33)	162 (16.95)	0.351
Married, n(%)	91 (73.98)	651 (68.10)	0.185
Duration of hypertension (years), median (range)	5 (3–38)	5 (3–63)	0.444
Health care provision capacity-high, n(%)	66 (53.66)	500 (52.30)	0.777
Hypertension intervention type			<0.001
No intervention, n(%)	1 (0.08)	300 (31.38)	-
One intervention, n(%)	42 (34.15)	243 (25.42)	-
Two interventions, n(%)	80 (65.04)	413 (43.20)	-
Physical functioning, median (range)	55 (0–100)	70 (0–100)	<0.001
Role-physical, median (range)	0 (0–100)	0 (0–100)	0.043
Body pain, median (range)	52 (10–100)	62 (0–100)	0.993
General health, median (range)	35 (0–92)	40 (0–100)	0.795
Vitality, median (range)	50 (10–85)	55 (10–90)	0.060
Social functioning, median (range)	62.5 (0–100)	75 (0–100)	0.018
Role-emotional, median (range)	0 (0–100)	0 (0–100)	0.001
Mental health, median (range)	52 (12–88)	56 (8–88)	0.029
Reported Health Transition, median (range)	50 (25–100)	50 (0–100)	0.161
PCS, median (range)	38.75 (3–98)	42.75 (0–99.25)	0.053
MCS, median (range)	45 (9.75–90.25)	53 (4.50–94.5)	0.003

### Propensity score analysis

After propensity score matching, two patients in the high continuity of care group and 93 in the low continuity of care group were off common support. Therefore, 121 patients in the high continuity of care group and 863 in the low continuity of care group were identified. The variables used in the logistic regression analysis are presented in Table 2. The final model accurately differentiated between the treated and control groups, with an area under the curve (AUC) of 0.7519; this area was well calibrated as the Pearson *χ* ^2^ test confirmed a good fit of the logistic regression = 533.96, *p* = 0.53.

**Table 2:** The associations between variables used to match with propensity score in logistic regression

Variable	Coefficient	Standard error	Z	*p*	95 % confidence Interval	
Age						
[60,70)	−0.140	0.305	−0.461	0.645	−0.737	0.457
[70,80)	0.029	0.304	0.095	0.924	−0.566	0.624
[80,108)	0.127	0.384	0.331	0.741	−0.625	0.879
Female gender	−0.113	0.215	−0.524	0.600	−0.534	0.309
Married	−0.318	0.244	−1.310	0.192	−0.796	0.160
Higher than primary educational level	0.105	0.269	0.388	0.698	−0.423	0.633
Duration of hypertension	−0.025	0.024	−1.060	0.291	−0.072	0.022
Type of township health center						
Type 2	0.825	1.120	0.734	0.463	−1.380	3.030
Type 3	3.650	1.020	3.590	0.000	1.650	5.640
Type 4	3.710	1.020	3.640	0.000	1.710	5.710
Type 5	3.010	1.020	2.930	0.003	0.998	5.010

After matching, the samples were considered sufficiently balanced: Rubins’ B = 10.3 and Rubin’s R = 0.89 [41]. Besides, no major differences were observed regarding baseline characteristics for the high continuity of care group and the low continuity of care group for all *p* values with differences > 0.1. Therefore, variables were satisfactorily balanced.

### DID analysis of HRQoL

As presented in Table 3, the results of kernel-based propensity score matching DID analysis for PCS and MCS before and after the observation period showed that patients in the high continuity of care group reported substantial improvements in PCS (DID = 5.192 ± 1.970) and MCS (DID = 7.900 ± 1.815). Furthermore, the improvements were statistically significant (all *p* < 0.05) when comparing the high and the low continuity of care groups. In the eight dimensions of HRQoL, we found that patients in the high continuity of care group reported better improvement in all dimensions except for body pain (DID = −3.392 ± 2.071). The differences of improvement in these domains were statistically significant: physical functioning, role-physical, general health, role-emotional, and mental health. Additionally, no substantial evidence demonstrated that better continuity of care could improve reported health transition (DID = 0.684 ± 1.45, *p* = 0.744 > 0.05).

**Table 3:** Results of kernel-based propensity score matching DID models with HRQoL

Variable	Pretest				Posttest				Diff-in-Diff	*p*
	High continuity of care group	Low continuity of care group	Diff	*p*	High continuity of care group	Low continuity of care group	Diff	*p*		
Physical functioning	56.488	65.09	−8.602	<0.001	71.364	69.092	2.271	0.157	10.873	<0.001
Role-physical	21.074	29.667	−8.593	0.001	43.388	43.911	−0.522	0.842	8.070	0.030
Body pain	57.521	56.860	0.661	0.652	62.595	65.326	−2.731	0.062	−3.392	0.102
General health	38.851	39.852	−1.001	0.543	49.355	45.142	4.213	0.011	5.214	0.025
Vitality	50.124	54.852	−4.728	<0.001	58.781	60.873	−2.092	0.072	2.636	0.109
Social functioning	63.165	67.132	−3.967	0.005	73.864	74.905	−1.042	0.461	2.925	0.143
Role-emotional	30.328	41.668	−11.340	<0.001	64.463	56.857	7.606	0.006	18.946	<0.001
Mental health	51.198	55.829	−4.631	<0.001	65.322	62.861	2.461	0.025	7.092	<0.001
Reported Health Transition	59.298	55.351	3.947	0.878	53.926	49.295	4.631	0.002	0.684	0.744
Physical component summary	43.483	47.867	−4.384	0.002	56.676	55.868	0.808	0.562	5.192	<0.001
Mental component summary	48.704	54.870	−6.166	<0.001	65.607	63.874	1.733	0.177	7.900	0.008

## Discussion

In the present study, we showed that continuity of care had a positive effect on HRQoL. Hypertensive patients who had been treated by the same physician for the past 2 years had better quality of life both physically and emotionally compared with those treated by several physicians. However, regarding the eight subscales of SF-36, patients reported positive effects of continuity of care on physical functioning, role-physical, general health, role-emotional as well as mental health; these effects were statistically significant. However, no significant differences were found on reported health transition.

Some studies, as did ours, have attempted to establish a relationship between continuity of care and HRQoL. The results of the present study differed from those reported by Hanninen and Gulliford [21, 22]. We showed that good continuity of care could lead to better scoring in both PCS and MCS of the SF-36. A similar result was demonstrated by Hanninen, who found that good continuity of care was significantly associated with the better well-being dimensions of the SF-20. However, the continuity of care effects on the eight HRQoL dimensions observed in the present study differed from the findings of the other two studies in that we found positive effects on physical functioning, role-physical, general health, role-emotional as well as mental health. In contrast, Hanninen found positive effects on body pain and social functioning. Contrary to our findings and those by Hanninen, Gulliford did not find an association between continuity of care and SF-12 PCS or MCS.

For the reasons stated above, the results from these three studies are not directly comparable. First, the three studies focused on different medical conditions. Hanninen’s and Gulliford’s studies focused on patients with diabetes, whereas the present study focused on patients with hypertension. Second, the current study used the binary variable of whether hypertensive patients were treated by the same physician or different ones to measure continuity of care, which is similar to the method used by Hanninen but different to that used by Gulliford. The latter study was based on a new questionnaire designed to measure the experienced continuity of care for type 2 diabetes (Experienced Continuity of Care—Diabetes Mellitus [ECC-DM]). Therefore, to improve the comparability of different studies, more unified measurement tools are needed to evaluate continuity of care for specific medical conditions.

Most of the previous studies used claims data from health insurance reimbursement databases [3–6, 11, 42]. Moreover, the outcomes of these studies focused on health resource utilization (hospitalizations and emergency department visits) and healthcare expenses. As Bentler [43] demonstrated, claim-based continuity of care measures cannot reflect patient perceptions of continuous patient-provider relationships. Thus, he suggested that claim-based data should be incorporated with patient reports to fully evaluate continuity of care. We implemented a 2-year cohort study to obtain additional observational variables and reduce the confounding bias. Furthermore, selection bias is a significant problem when assessing the effect of continuity of care. Four related studies have considered this type of bias [13, 19, 22, 44], whereas the vast majority of previous studies have neglected it. In this study, selection bias was controlled via kernel-based propensity score matching.

Regarding the question of whether the basic public health care delivery system for patients with hypertension should be changed, the present research provided sound evidence. The present results showed that a long-term relationship between hypertensive patients and physicians could improve patient HRQoL. This shows the importance of family doctors for patients with hypertension. However, whether family doctors are of significance for patients with other conditions still needs further study. According to our knowledge, those who are willing to visit a fixed physician are more familiar with their physician and are more willing to accept their advice, resulting in better treatment compliance. The mediating effects among continuity of care, awareness, compliance, and outcomes (such as HRQoL, medical expenses, healthcare utilization) also need to be further tested.

This study is characterized by certain strengths. Instead of using claims data or cross-sectional investigation data separately, a cohort study was implemented. As a contribution of the follow-up service records, the recall bias was reduced and the calculation of continuity of care was ensured to be as accurate as possible. Additionally, propensity score matching was used to eliminate selection bias, which theoretically led to the reduction of the risks of baseline demographic distribution and other covariate differences. Furthermore, the use of a DID analysis minimized the potential differences for those patients in the high continuity of care and low continuity of care groups during the 2-year follow-up.

However, this current study also had certain limitations. First, as Robles [19] assumed, the use of health services may influence continuity of care. This variable was not used as a covariate to estimate the propensity score in this study because data collection proved to be difficult. Second, as classified by Jee [45], continuity of care was calculated primarily based on duration of provider relationship, density of visits, dispersion of providers, sequence of providers, and subjective estimates. In the present study, although the fixed physician and patient relationship was measured, it could not represent all dimensions of continuity of care. Third, the cohort study was implemented only in Qianjiang District, which does not represent all settings in China. Future studies should include more study settings to yield stronger evidence.

## Conclusion

A long-term physician-patient relationship may improve the HRQoL of patients with hypertension. Patients with hypertension and good continuity of care presented significant improvements in physical quality of life, emotional quality of life, and some dimensions of HRQoL such as physical functioning, role-physical, general health, and role-emotional as well as mental health. Measurement tools that are more unified are needed to evaluate continuity of care in further studies.
